# Introducing the “AI Language Models in Health Care” Section: Actionable Strategies for Targeted and Wide-Scale Deployment

**DOI:** 10.2196/53785

**Published:** 2023-12-21

**Authors:** Alexandre Castonguay, Christian Lovis

**Affiliations:** 1 Faculté des sciences infirmières Université de Montréal Montréal, QC Canada; 2 Faculty of Medicine University of Geneva Geneva Switzerland

**Keywords:** generative AI, health care digitalization, AI in health care, digital health standards, AI implementation, artificial intelligence

## Abstract

The realm of health care is on the cusp of a significant technological leap, courtesy of the advancements in artificial intelligence (AI) language models, but ensuring the ethical design, deployment, and use of these technologies is imperative to truly realize their potential in improving health care delivery and promoting human well-being and safety. Indeed, these models have demonstrated remarkable prowess in generating humanlike text, evidenced by a growing body of research and real-world applications. This capability paves the way for enhanced patient engagement, clinical decision support, and a plethora of other applications that were once considered beyond reach. However, the journey from potential to real-world application is laden with challenges ranging from ensuring reliability and transparency to navigating a complex regulatory landscape. There is still a need for comprehensive evaluation and rigorous validation to ensure that these models are reliable, transparent, and ethically sound. This editorial introduces the new section, titled “AI Language Models in Health Care.” This section seeks to create a platform for academics, practitioners, and innovators to share their insights, research findings, and real-world applications of AI language models in health care. The aim is to foster a community that is not only excited about the possibilities but also critically engaged with the ethical, practical, and regulatory challenges that lie ahead.

## The Promise and Potential of AI in Health Care

In this editorial, we introduce a new section in *JMIR Medical Informatics*, “AI Language Models in Health Care,” dedicated to exploring the transformative potential of generative artificial intelligence (AI) and the developments of cognitive AI in the health care industry. At its current stage of development, AI has already shown the potential to guide human interactions, facilitate understanding, and foster respectful communication. Most of all, with the phenomenon surrounding the global release of ChatGPT [1], it is the first time that information technology has demonstrated the ability to leverage the informational content of texts and human narratives in such a substantial way. In a world where over 65% of the population is connected to the internet [2], this potential is substantial and wide-reaching.

Within the health care sector specifically, AI and large language models in particular have progressed toward fulfilling their ambitious promises. They have delivered concrete outcomes such as improving decision-making processes at all levels of influence including for patients, clinicians, health systems, and society as a whole [3]. Yet, the journey of generative AI is still nascent. While the rapid adoption of generative AI indicates its potential, concerns regarding its accuracy and preparedness in managing its associated risks remain [4]. Additionally, being in its early stages of deployment, the long-term positive impacts are anticipated but not yet fully realized or evidenced.

## The Aim of This New Section

In this new journal section, we focus on generative models and how they can redefine the boundaries of health care, all while acknowledging the challenges and limitations. By spotlighting generative models, we hope to illuminate their potential while realistically addressing the challenges associated with their implementation in diverse care settings.

The objective of this section is to highlight groundbreaking innovations empowered by AI language models with a keen focus on ensuring their safe, effective, and enduring implementation. We seek to delve into solid actionable strategies for implementation at various scales of deployment and sustainability, striving to facilitate the swift yet secure integration of these technologies into health care environments. Our ambitions extend beyond theoretical contributions; we seek mature, ready-to-implement initiatives that not only break new ground but also offer clear actionable strategies for wide-scale deployment. The goal is to leapfrog over efforts that should have been made 20 years ago and to streamline the integration process of innovations that will propel the health care sector 20 years into the future.

This section is dedicated to the implementation of AI language models in health care and focuses on prioritizing efforts that highlight the best and most accessible opportunities. Its goal is to leverage emerging technologies while aiming to deliver substantial benefits to as many people as possible.

By featuring papers with clear detailed methodologies, we aim to inspire and facilitate the adaptation and replication of these innovations across diverse health care environments. In doing so, we hope that this new section serves not only as a road map but also as an inspiration, catalyzing the ambition to redefine the limits of health care through the responsible strategic use of AI. More information on suggested topics for submission can be found in the corresponding section of this editorial.

## The Nuanced Role of Generative AI in Health Care

While digitalization remains the foundation upon which our current health care advancements are built [5], it is essential to differentiate between the overarching digital transformation and the nuanced capabilities of generative AI. Generative AI models, a subset of the broader AI landscape, hold unique promise and challenges for the health care sector. Unlike traditional AI systems that operate within the confines of given data sets, generative AI can create new, unseen content [6], enhancing clinical decision-making, patient interactions, and even medical research [3]. This ability to generate information places a heightened responsibility on ensuring the reliability, accuracy, and ethics of its outputs. As we navigate the vast world of health care AI, it is imperative to hone our focus on these generative models and their distinct potential to reshape patient care, medical education, and health system operations. By emphasizing their role, we aim to highlight not only their innovative capabilities but also the accompanying challenges that need to be addressed for a meaningful and safe integration into health care environments.

## Current State of Affairs: The Great Paradox

Despite significant advancements in AI often leaving populations worldwide in awe, many areas within the health care sector still notoriously fail to impress, particularly in terms of digital health standards, equitable access, and system interoperability [7]. Some medical sectors in specific places are at the forefront of scientific and technological advances, but most countries or fields like long-term care lag, trapped in the digital health standards of the 1970s. The major challenges are not the use of faxes or digital tools but rather the equity of access for society and interoperability for the most advanced systems.

As a sector receiving substantial investment, being the second-largest spending category among Organisation for Economic Co-operation and Development countries, the health care industry is more than a line item in a budget [8]. Fundamentally, it is charged with the critical task of promoting and safeguarding health—one of the indispensable pillars supporting societal stability on which all stands. Without it, everything else teeters on the edge of collapse.

The guiding principle of health care has long been “Primum non noccere,” which translates to “First, do no harm.” Yet in today’s rapidly evolving digital landscape, the traditional interpretation of this maxim can no longer serve as an excuse for inaction. In our current state, maintaining the status quo or doing nothing is, paradoxically, causing harm.

## The Call to Action: Leading the Digital Charge

Digitalization, with its foundational role in ensuring data interoperability, has enabled the rise of AI in health. In turn, AI has provided a platform for the advent of advanced generative models. Today, amid this AI revolution, it is striking and unacceptable that, despite its crucial role and significant investment, health care often finds itself trailing [9,10]. As the technological landscape evolves at an unprecedented pace, we should not merely object to this essential industry remaining technologically outdated; we should insist that it leads the charge, setting the pace rather than lagging. However, this is not just about technological advancements; it is about the essence of care.

The potential of generative models in health care is vast, and our discussion should reflect an informed enthusiasm. Recognizing the capabilities and promise they bring does not mean we are overlooking the complexities. It means we are optimistic about what can be achieved without being naive about the challenges. While we push for innovation in health care, our insistence is on not only digital advancements but also meaningful, responsible, and nuanced progression. Now more than ever, advancing with clarity and purpose is the only way health care can genuinely uphold “Primum non noccere” in this digital age.

## The Present Challenges: Strategies for Effective Implementation

A wealth of digital health solutions already exists [11], each seemingly more promising than the last. However, as technology advances, understanding these solutions requires increasingly specialized skills.

For instance, when users turn to chatbots such as ChatGPT for specific health care queries—be it programming a medical device or interpreting a health symptom—the ideal response from such systems should be precise, accurate, and aware of its limitations. However, instead of straightforward confessions like, “I’m unfamiliar with that programming language” or “That symptom is beyond my knowledge,” these models might sometimes fabricate answers to feign understanding [12]. This tendency to generate unverified responses, without a built-in “truth threshold,” not only risks misinformation but also erodes trust in these technologies in a field where clarity is paramount.

This situation underscores the imperative for the industry to enhance the reliability and transparency of such tools and, equally, for users to be aptly equipped to use and interpret these advanced technologies. They must act as a “truth threshold” for themselves, discerning the reliability of the information provided.

This represents an indispensable uphill battle to evaluate and prioritize solutions for large-scale implementation since the need has never been greater for efficient, safe, and intelligent solutions that improve performance and quality of life for patients and health care professionals while benefiting institutions and the health care system as a whole.

## The Journey Forward: Meticulous Implementation and Sustainability

In light of AI’s transformative potential—and recognizing it both as a beacon of groundbreaking advancements and, if unchecked, as one of the most significant potential threats to humanity—our call to action underscores the great importance of not only progressing AI’s technical development and governance. It is, for example, equally crucial to address the domains of regulatory constraints, interoperability, accountability, and liability with utmost diligence [13]. Drawing a parallel from the aeronautics industry, where manufacturers, air traffic control, and stringent flight regulations collectively ensure some of the safest travel experiences [14], we believe that the journey of AI integration in health care must adopt a similar, if not greater, level of care and rigor.

However, it is pivotal to highlight that sheer development and regulations will not suffice. The essence of successful innovation lies in its effective implementation. This includes rigorous preparation, meticulous execution, and sustained support to ensure both immediate success and long-term sustainability. Given the health care industry’s paramount role in life preservation and its significant costs, it is imperative that it not only aligns with technological advancements but also leads them. Leading does not mean merely developing new solutions but also ensuring they are seamlessly integrated and enduringly effective. The health care sector should be at the helm of innovation, shaping the future and guaranteeing that the innovations are not only introduced but also enduringly implemented for the benefit of all.

## Suggested Topics for Submission

This section provides a list of example topics tailored to align with our broad focus on the successful implementation of AI language models in diverse health care settings. We encourage contributors to delve into the process, use, outcomes, and influential factors of AI integration, aiming for a holistic and practical discourse. The topics listed are not exhaustive, and we welcome diverse perspectives and innovative approaches.

- Comparative analyses of policies and regulations: Explore the varying landscapes of AI regulation in health care across different countries. Delve into how specific policies either facilitate or hinder the implementation of AI language models, providing insights into creating conducive environments for global adoption.
- Strategies for reliability, transparency, and ethical use: Discuss and develop strategies to ensure the ethical, transparent, and reliable application of AI language models in health care. Share best practices, guidelines, and frameworks that help in establishing trust among stakeholders and ensuring patient safety.
- Comprehensive methodologies for implementation: Provide detailed blueprints and methodologies for the successful integration of AI language models in health care settings. Focus on the entire life cycle, from initial planning and integration to long-term management, ensuring applicability for researchers, industry professionals, policy makers, and decision makers.
- Case studies on real-world impact and effectiveness: Share real-world examples illustrating the impact of AI language models on health care delivery. Highlight both successes and challenges, discussing the tangible outcomes, lessons learned, and strategies for overcoming obstacles.
- Evaluations of outcomes and impact on health care delivery: Conduct thorough evaluations of AI language model implementations, examining a wide range of outcomes including acceptability, adoption, cost-effectiveness, and impact on health care efficiency and patient satisfaction. Explore how these technologies contribute to or challenge the equity, timeliness, and patient-centeredness of health care services.

By addressing these topics, authors contribute to a collaborative, rigorous, and transformative discourse, propelling the health care sector toward seamless integration of AI language models and ensuring a lasting positive impact on global health care delivery.

## Abbreviations

AI: artificial intelligence

## References

[1] Hu K (2023). ChatGPT sets record for fastest-growing user base - analyst note. Reuters.

[2] (2023). Population of global offline continues steady decline to 2.6 billion people in 2023. ITU.

[3] Thirunavukarasu A, Ting DSJ, Elangovan K, Gutierrez L, Tan TF, Ting DSW (2023). Large language models in medicine. Nat Med.

[4] Chui M, Yee L, Hall B, Singla A, Sukharevsky A (2023). The state of AI in 2023: generative AI’s breakout year. McKinsey & Company.

[5] (2023). WHO launches a new Global Initiative on Digital Health supported by the G20 Presidency. World Health Organization.

[6] Russell S, Norvig P (2020). Artificial Intelligence: A Modern Approach, 4th edition.

[7] Paik K, Hicklen R, Kaggwa F, Puyat C, Nakayama L, Ong B, Shropshire JNI, Villanueva C (2023). Digital determinants of health: health data poverty amplifies existing health disparities-a scoping review. PLOS Digit Health.

[8] (2023). General government spending. OECD Data.

[9] Cam A, Chui M, Hall B (2019). Global AI survey: AI proves its worth, but few scale impact. McKinsey & Company.

[10] Goldfarb A, Teodoridis F (2022). Why is AI adoption in health care lagging?. Brookings.

[11] (2023). Classification of digital interventions, services and applications in health: a shared language to describe the uses of digital technology for health, 2nd ed. World Health Organization.

[12] Alkaissi H, McFarlane SI (2023). Artificial hallucinations in ChatGPT: implications in scientific writing. Cureus.

[13] Working Group on Digital and AI in Health (2020). Reimagining global health through artificial intelligence: the roadmap to AI maturity. Broadband Commission.

[14] (2023). Global Aviation Safety Plan 2023–2025. International Civil Aviation Organization.

